# Exposure to Mouse Allergen in U.S. Homes Associated with Asthma Symptoms

**DOI:** 10.1289/ehp.11847

**Published:** 2008-10-06

**Authors:** Päivi M. Salo, Renee Jaramillo, Richard D. Cohn, Stephanie J. London, Darryl C. Zeldin

**Affiliations:** 1 National Institute of Environmental Health Sciences, National Institutes of Health, Department of Health and Human Services, Research Triangle Park, North Carolina, USA; 2 Constella Group, LLC, Durham, North Carolina, USA

**Keywords:** allergy, asthma, exposure, indoor, mouse allergen

## Abstract

**Background:**

Most studies investigating the role of residential mouse allergen exposures in asthma have focused on inner-city populations.

**Objective:**

We examined whether elevated mouse allergen levels were associated with occupants’ asthma status in a nationally representative sample of U.S. households.

**Methods:**

Data for this study were collected as part of the National Survey of Lead and Allergens in Housing. This cross-sectional study surveyed 831 housing units inhabited by 2,456 individuals in 75 different locations throughout the United States. The survey obtained information on demographics, household characteristics, and occupants’ health status by questionnaire and environmental observations. We used a polyclonal immunoassay to assess concentrations of mouse urinary protein (MUP) in vacuumed dust collected from various indoor sites.

**Results:**

Of the surveyed homes, 82% had detectable levels of MUP, and in 35% of the homes, MUP concentrations exceeded 1.6 μg/g, a level that has been associated with increased mouse allergen sensitization rates. Current asthma, defined as having doctor-diagnosed asthma and asthma symptoms in the preceding 12 months, was positively associated with increased MUP levels. The observed association was modified by atopic status; in allergic individuals, elevated MUP levels (> 1.6 μg/g) increased the odds of having asthma symptoms [adjusted OR = 1.93; 95% confidence interval (CI), 1.14–3.27], but we found no association in those who did not report allergies (adjusted OR = 0.69; 95% CI, 0.33–1.44).

**Conclusions:**

In allergic asthma, residential mouse allergen exposure is an important risk factor for asthma morbidity.

Mouse allergen exposure is a well-recognized risk factor for allergic sensitization and asthma in occupational settings (Bush and Stave 2003; Phipatanakul 2002). More recently, it has also been identified as a factor that may contribute to asthma morbidity in nonoccupational populations. Exposure and sensitization to mouse allergen are common, particularly among asthmatic populations in inner cities (Phipatanakul et al. 2000a, 2000b). In the National Cooperative Inner-City Asthma Study (NCICAS), which was the first study to examine mouse allergen exposures in residential environments, mouse allergen was detectable in an extraordinarily high percentage of the homes (74–100%) (Phipatanakul et al. 2000a). Findings from previous studies suggest that sensitization rates to mouse allergen increase with increasing exposure levels, although the dose–response relationship may not be linear (Matsui et al. 2007). Reported sensitization rates have generally ranged from 7% to 18% (Matsui et al. 2005; Phipatanakul et al. 2000b, 2007), although even higher rates have been reported (up to 40%) among asthmatic children with high exposure levels (Matsui et al. 2007). Recent studies have also demonstrated that elevated mouse allergen levels are associated with asthma morbidity in inner-city children and adults, highlighting the importance of mouse allergen in this population (Chew et al. 2006; Matsui et al. 2006).

Although most studies that have been published have targeted inner-city populations (Chew et al. 2003, 2006; Matsui et al. 2005, 2006, 2007; Phipatanakul et al. 2000a, 2000b), mouse allergen exposure may not be restricted to urban populations that have disproportionately high asthma prevalence rates. Studies have shown that mouse allergen exposure and mouse sensitivity can be surprisingly widespread, even outside inner-city areas (Matsui et al. 2004; Phipatanakul et al. 2005). Yet the role of residential mouse allergen exposure in asthma remains poorly characterized among the general U.S. population because previous studies have focused mainly on selected populations (i.e., children, asthmatics, and urban residents).

The National Survey of Lead and Allergens in Housing (NSLAH) was the first population-based study to estimate mouse allergen levels and examine mouse allergen exposure in relation to asthma in a nationally representative sample of the U.S. households (Vojta et al. 2002). We have previously described the details of the exposure characteristics (Cohn et al. 2004). In this article, we report associations between mouse allergen exposures and asthma-related symptoms in the study population.

## Materials and Methods

### Study design and procedures

The NSLAH was a cross-sectional study that used a complex, multistage design to sample the U.S. population of permanently occupied, noninstitutional housing units that permit resident children. The survey was approved by the National Institute of Environmental Health Sciences Institutional Review Board in 1998, and all study procedures complied with applicable regulations. A detailed description of the study design and methodology can be found elsewhere (Vojta et al. 2002). Briefly, the survey examined a nationally representative sample of 831 housing units, inhabited by 2,456 individuals, within 75 different locations throughout the United States. After an adult representative of the household gave written informed consent, information on demographics, household characteristics, and occupants’ health status was collected by questionnaire. During the home visit, environmental data were also acquired by sample collection and inspection of the housing unit.

### Assessment of asthma-related outcomes

The resident questionnaire obtained information on doctor-diagnosed asthma and allergies, wheezing, asthma symptoms in the preceding year, and current asthma medication use. In this survey, an adult respondent identified individual household members who had doctor-diagnosed asthma, including adults with childhood-onset asthma. Current asthma, which was our primary outcome measure, was ascertained with a question confirming asthma symptoms in the past year. Atopic status was assessed by report of physician diagnosis of allergies (e.g., hay fever, skin, or food allergies); subjects with reported physician-diagnosed allergies were classified as atopic, whereas subjects without reported allergies were considered nonatopic in the analyses.

### Environmental sampling

Single surface dust samples were collected from a bed, from a sofa or a chair, and from bedroom, living room, and kitchen floors, as previously described (Vojta et al. 2002). Each sampling site was vacuumed for 5 min using a Eureka Mighty-Mite 7.0-A vacuum cleaner (Eureka Company, Bloomington, IL). We measured concentrations of mouse allergen [mouse urinary protein (MUP)] in dust with a competitive inhibition enzyme-linked immunosorbent assay using purified antigen and a polyclonal anti-MUP antibody (Greer Laboratories, Inc., Lenoir, NC). The lower limit of detection (LOD) was 0.004 μg/mL. Because of differences in extraction concentrations, the LOD for MUP per gram of dust varied slightly, being 0.25 μg/g for most of the samples (Cohn et al. 2004; Vojta et al. 2002).

### Statistical analyses

For the statistical analyses, we log-transformed MUP concentrations because of skewed distributions. In addition to the site-specific concentrations (*n* = 5), we calculated a house index (i.e., the mean of all measured sampling location concentrations) to represent the average MUP concentration in the household. In 17.7% of the households, MUP levels were lower than the LOD. To maximize the number of samples in the analysis, we assigned samples with concentrations less than the LOD to one-half of the value of the LOD. Samples that had insufficient amount of dust for the allergen analysis were considered missing. Allergen measurements were available from at least one room for 99.2% (*n* = 824) of the homes. For the site-specific analyses, the corresponding percentage varied from 78.9% to 88.5%, being lowest for living room upholstery and highest for kitchen floors.

We calculated odds ratios (ORs) with 95% confidence intervals (CIs) for the asthma-related outcomes using logistic regression. We excluded from the analyses subjects with missing data on the exposures and covariates, leaving 2,028 of 2,456 subjects in the analysis (83%). The characteristics of the excluded subjects did not differ from those included in the analyses [see Supplemental Material, Table 1 (http://www.ehponline.org/members/2008/11847/suppl.pdf)]. The models we present here are adjusted for age, sex, race, education, smoking, and survey season. We used household-level data (indoor smoking in the home) to assess smoking exposure because the survey did not obtain information on personal smoking. Adjusting for other social and household factors (e.g., income, housing type) did not change the ORs appreciably [< 10%; see Supplemental Material, Table 2 (http://www.ehponline.org/members/2008/11847/suppl.pdf)]. We also examined whether the effect estimates were influenced by dust weight and presence of other indoor allergens [*Dermatophagoides farinae* 1 (Der f 1, dust mite allergen), *Dermatophagoides pteronyssinus* 1 (Der p 1, dust mite allergen), *Blattella germanica* 1 (Bla g 1, cockroach allergen), *Felis domesticus* 1 (Fel d 1, cat allergen), *Canis familiaris* 1 (Can f 1, dog allergen), and *Alternaria alternata* (mold)] and bacterial lipopolysaccharide (endotoxin). Because the observed association was modified by atopic status, we present separate ORs for atopic and nonatopic individuals.

We conducted logistic modeling using SUDAAN (version 9.0; RTI International, Research Triangle Park, NC), and we used Taylor series linearization methods to adjust standard errors for the complex survey design. We applied sample weights to all estimates to account for housing selection probabilities, nonresponse, and poststratification. The SUDAAN software also took into account effects of clustering in the data, including multiple occupants in the same household. Details of statistical weighting for the NSLAH can be found elsewhere (Vojta et al. 2002). We further characterized the relationship between current asthma and MUP concentrations by generalized additive models (GAMs). We used the gam( ) function of the GAM package in R software (version 2.4.0, open source: http://cran.r-project.org/) to fit the models and graphed the fitted model relationships (R Foundation 2008).

## Results

Table 1 presents the weighted characteristics of the study population. The characteristics of the survey sample, including distributions of housing characteristics, socioeconomic, and demographic factors, were very similar to characteristics obtained from other national surveys (Vojta et al. 2002). The prevalence of asthma was comparable with other national estimates (Centers for Disease Control and Prevention 2006). The lifetime prevalence of doctor-diagnosed asthma was 11.2%, and 6.9% of the study subjects reported active asthma symptoms in the past 12 months. Most of the current asthmatics (77%) reported doctor- diagnosed allergies and used asthma medication (71%). Recent wheezing was more commonly reported than were asthma symptoms and did not differ from other national estimates (Arif et al. 2003; Eldeirawi and Persky 2004).

Exposure distributions are summarized in Figure 1 of the Supplemental Material (http://www.ehponline.org/members/2008/11847/suppl.pdf). Most of the homes (82%) had detectable levels of MUP. In 35% of the homes, MUP concentration in at least one site in the household exceeded 1.6 μg/g, a level that has been associated with increased mouse allergen sensitization rates (Phipatanakul et al. 2000b). Of the sampled sites, kitchens had highest concentrations (geometric mean = 0.52 μg/g) and living room upholstery had lowest concentrations (geometric mean = 0.28 μg/g) of MUP. Elevated MUP levels were most prevalent in multifamily homes (e.g., high-rise apartments) and mobile homes, older homes, and low-income homes. More detailed information on exposure characteristics has been published elsewhere (Cohn et al. 2004).

Prevalence of current asthma was significantly higher if MUP levels exceeded 1.6 μg/g in any location in the home (Table 2). Table 3 shows unadjusted and adjusted effect estimates for the association between current asthma and elevated MUP levels. Table 4 presents ORs for atopic and nonatopic individuals separately because atopic status modified the observed association. In atopic individuals, elevated levels of MUP significantly increased the odds of having asthma symptoms in the past year (adjusted OR = 1.93; 95% CI, 1.14–3.27). We found no association in nonatopic individuals (adjusted OR = 0.69; 95% CI, 0.33–1.44). After adjusting for potential confounders, including other indoor allergens, endotoxin, or dust weight, the magnitude of the effect did not change appreciably (Table 4). Consistent with these results, asthma medication use—which often suggests persistent and/or more severe asthma—was also positively associated with elevated MUP levels among atopic individuals (adjusted OR = 1.86; 95% CI, 1.03–3.36).

**Figure 1:**
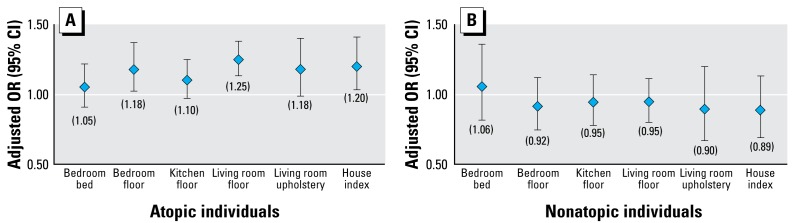
Adjusted ORs and 95% CIs for the association between current asthma and MUP concentration in the household (continuous variable, logistic regression). The house index (mean of site-specific concentrations) and site-specific ORs correspond to a 2-fold increase in MUP concentration adjusting for age, sex, race, education, smoking, and survey season. The ORs for atopic (*A*) and nonatopic (*B*) individuals are presented separately (in parentheses) because the observed association was modified by atopic status.

**Figure 2:**
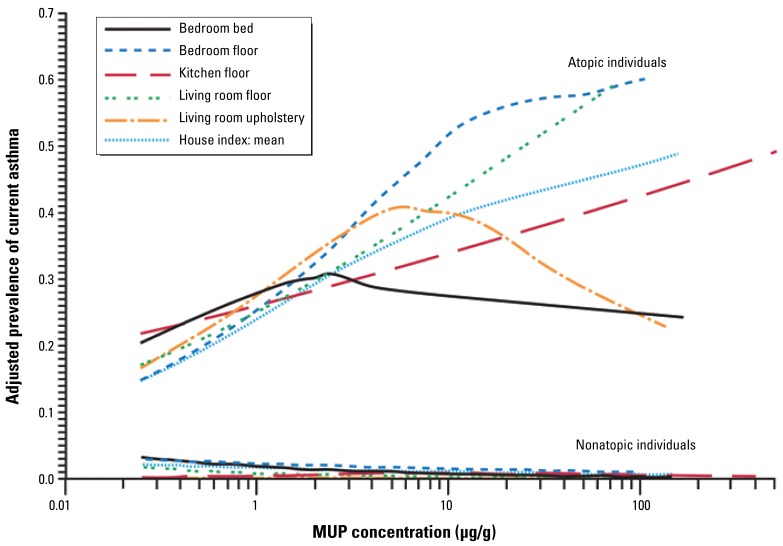
Smoothed plots showing adjusted prevalence of current asthma by MUP concentration for the house index and each sampling location. The estimated prevalence is adjusted for age, sex, race, education, smoking, and survey season.

To further characterize the relationship between current asthma and MUP levels in the home, we modeled the allergen concentration as a continuous variable. Figure 1 presents results from logistic regression analysis. The adjusted ORs for current asthma correspond to a 2-fold increase in MUP concentrations (site-specific and average concentration in the household). Complementary to logistic regression, we modeled the association using GAMs. The modeled relationships display trends in current asthma prevalence across MUP concentrations. The smooth plots (Figure 2) illustrate adjusted prevalence for the average and site-specific concentrations. Our findings suggest that prevalence of current asthma increases with increasing MUP concentrations, although the results were not as consistent for bedroom bed and living room upholstery as for the other sites. We found no clear indication of a threshold below which there was no increase in prevalence.

Wheezing was not significantly associated with elevated MUP levels in the total study population (Table 2). Most of those (71.4%) who reported either wheezing in the past month or in the past year did not report asthma symptoms. Because diseases other than asthma [e.g., chronic obstructive pulmonary disease (COPD)] might have contributed to wheezing, we conducted additional analysis in a subpopulation of individuals < 40 years of age (*n* = 1,541). In this subpopulation, wheezing is less likely to be associated with COPD because the bulk of the disease occurs in older age groups (Stang et al. 2000). In the younger subpopulation, recent wheezing was more prevalent among subjects who had elevated MUP levels in their homes than among those who did not (25.1% vs. 17.8%; *p* = 0.07 for difference). Table 3 of the Supplemental Material (http://www.ehponline.org/members/2008/11847/suppl.pdf) shows ORs for recent wheezing (in the past month and/or in the past year) among the younger age groups. Our results suggest that the association is modified by atopic status. After adjusting for potential confounders, elevated MUP levels in any location in the home significantly increased the odds of recent wheezing among atopic individuals (adjusted OR = 2.83; 95% CI, 1.35–5.96), but we found no association in nonatopic individuals (adjusted OR = 1.34; 95% CI, 0.75–2.39).

## Discussion

The NSLAH was the first study to examine the role of residential mouse allergen exposures in relation to asthma in nationally representative sample of U.S. households. We found that elevated levels of mouse allergen significantly increased the likelihood of having asthma-related symptoms among atopic individuals. In contrast, we found no association in nonatopic individuals. The association remained consistent after adjusting for potential confounders. Therefore, our findings suggest that mouse allergen exposure in the home is an important risk factor for allergic asthma and contributes independently to asthma morbidity among allergic individuals.

In allergic asthma, allergens play a key role in triggering and exacerbating asthma symptoms (Langley et al. 2003; Nelson 2000). Because people spend most of their time indoors, especially at home, allergen exposures in the home environment are of great importance in relation to asthma (Leech et al. 2002). Although mouse allergen is a well-recognized and widely studied allergen in occupational settings (Bush and Stave 2003; Phipatanakul 2002), it has only recently identified as a household allergen that may influence asthma morbidity in domestic settings. To date, most research has focused on inner-city homes in which mouse allergen has been found to be ubiquitous (Chew et al. 2003, 2006; Matsui et al. 2005, 2006, 2007; Phipatanakul et al. 2000a, 2000b). The results from the NSLAH, however, suggest that the presence of mouse allergen in U.S. homes is surprisingly common even outside inner-city environments. Although exposure levels tend to be higher in low-income, urban neighborhoods (Matsui et al. 2004; Simons et al. 2007), our findings demonstrate that elevated levels are not restricted to those environments. However, restricting the analysis to low-income, urban homes in the NSLAH, the prevalence and distributions of mouse allergen compared well with previous findings from inner-city populations. For example, in the NCICAS and NSLAH, 95% of low-income urban homes had detectable mouse allergen levels in at least one room. Detectable levels of mouse allergen in kitchens were also comparable (83% in the NSLAH; 87% in the NCICAS). However, the prevalence of elevated mouse allergen levels (> 1.6 μg/g) in kitchens was higher in the NCICAS than in the NSLAH (50% in the NCICAS; 33% in the NSLAH) (Cohn et al. 2004; Phipatanakul et al. 2000a). On the other hand, some studies in low-income urban populations have observed lower mouse allergen levels than those found in the NCICAS (Chew et al. 2006).

In the NSLAH, elevated mouse allergen levels were associated with asthma-related outcomes. After adjusting for potential confounders, including the presence of other indoor allergens, endotoxin, or dust weight, elevated levels of mouse allergen (> 1.6 μg/g) in the home increased the odds of having asthma symptoms in the past year approximately 2-fold. However, the observed association was modified by atopic status; we found the association in atopic but not in nonatopic individuals. Because elevated mouse allergen levels have been associated with several other environmental exposures, particularly exposures that relate to poor housing conditions (Simons et al. 2007), we cannot exclude the possibility that additional factors may have contributed to current asthma symptoms. Indeed, we have previously shown that *Alternaria* and endotoxin exposures contribute to asthma morbidity in this population (Salo et al. 2006; Thorne et al. 2005). However, our results suggest an independent association between asthma symptoms and mouse allergen levels because the observed association did not change appreciably after adjusting for potential confounders.

Although degree of atopy has been associated with mouse sensitivity and elevated mouse allergen levels among asthmatic inner-city children (Phipatanakul et al. 2000b), atopy per se was not associated with mouse allergen levels in the NSLAH. This may reflect differences in population characteristics: The NCICAS included only asthmatic children, whereas the NSLAH sample represented the general population. Studies have shown that the prevalence and degree of atopy tend to be higher among asthmatic inner-city children than among the general U.S. population (Arbes et al. 2005; Crain et al. 2002; Eggleston et al. 1998; Stevenson et al. 2001). Because we were not able to ascertain the specificity or degree of atopy in the NSLAH, atopy reflects subjects’ allergic status in general, not specific sensitization to mouse allergen. Although atopy is often confirmed by clinical measures (serum IgE, skin prick tests), the diagnosis of clinically relevant allergy also depends on symptom history. The presence of allergen-specific antibodies or a positive skin test response to a specific allergen does not necessarily mean that a patient has clinically significant symptoms when exposed to an allergen (Pastorello et al. 1995). However, questionnaire-based data may underestimate the prevalence of atopy because questionnaires are unlikely sensitive enough to detect all individuals who are atopic (Lakwijk et al. 1998). We acknowledge that the lack of objective information on study subjects’ atopic status is a limitation, but findings from the NCICAS suggest that mouse allergen concentrations > 1.6 μg/g are associated with increased risk for IgE-mediated mouse sensitization.

Because the literature-derived cutoff point that we used to dichotomize the exposure variable is somewhat arbitrary, we further characterized the relationship between current asthma and mouse allergen levels by conducting additional statistical analyses, including modeling the exposure as continuous variable and using complementary modeling techniques. Our modeling results remained rather consistent regardless of the methods used. Most of the site-specific analyses supported the concept that the prevalence of current asthma increased with increasing mouse allergen concentrations, but the trend was less clear for bedroom bed and living room upholstery dusts. Although a recent study has suggested that the shape of the dose–response relationship between mouse allergen levels and allergen-specific sensitization may not be linear, particularly at higher mouse allergen levels (Matsui et al. 2007), it is unlikely that the nonlinear shape of the smooth curves is associated with this phenomenon. In fact, mouse allergen levels in bedroom bed and living room upholstery dusts were lower than in the other sites. Furthermore, these two sites had more missing observations than did floor dust samples, which may have influenced the modeling accuracy.

Wheezing was not significantly associated with elevated mouse allergen levels in the total study population. Although wheezing is a common symptom of asthma, a variety of respiratory disorders that lead to airway narrowing or obstruction can be associated with wheezing (Gong 1990). Because most of those who reported recent wheezing did not report asthma symptoms, we hypothesized that diseases other than asthma (e.g., COPD) might have contributed to wheezing. However, COPD is a slowly progressive disease and rarely occurs in individuals < 40 years of age (Halbert et al. 2003; Stang et al. 2000). When we restricted our analysis to a younger subpopulation (< 40 years of age), wheezing was strongly associated with elevated mouse allergen levels among atopic individuals. Consistent with reported asthma symptoms, we saw the strongest effects with elevated MUP levels in bedroom floor and living room dusts. We recognize that the wheezing-related results should be interpreted with some caution because we were not able to ascertain and differentiate whether wheezing resulted from asthma, COPD, or other respiratory disorders.

We acknowledge that our study has limitations. Because of the cross-sectional nature of the study, temporal relationships may be difficult to determine. Therefore, we focused mainly on asthma-related outcomes in the recent past. We lacked detailed sensitization data (e.g., skin prick test, specific IgE) but assessed atopy on self-reported physician-diagnosed allergies. Although airborne allergen concentrations are considered more relevant measures of personal exposure, large-scale epidemiologic studies traditionally use measurement of allergen concentration in reservoir dust as a surrogate measure of exposure, largely because of practical and financial reasons. In this study, we assessed mouse allergen levels in dust across multiple household sites, in order to characterize the exposure in detail. The NSLAH conducted sampling in each geographic region throughout seasons to mitigate any possible seasonal bias. Although the cutoff point that we used to dichotomize the exposure for modeling is somewhat arbitrary, findings from the NCICAS study have shown that mouse allergen concentrations above this threshold have been associated with an increased risk for IgE-mediated mouse sensitization (Phipatanakul et al. 2000b). Moreover, Matsui et al. (2005) have demonstrated that even with lower allergen concentrations in dust, mouse allergen is detectable in the vast majority of air samples; in their study, 90% of the participants with > 0.5 μg/g of mouse allergen in settled dust samples had detectable levels of airborne mouse allergen in their home.

The major strength of the study is its national representativeness. Vojta et al. (2002) showed that the demographic characteristics of the weighted survey sample did not differ appreciably from characteristics of other national surveys, including the 1995 and 1997 American Housing Survey and the 1998 and 1999 Current Population Survey. Furthermore, asthma prevalence rates compared well with other national estimates (Centers for Disease Control and Prevention 2006). The NSLAH was the first study to evaluate the importance of residential mouse allergen exposures among the general U.S. population.

In summary, this study suggests that mouse allergen is an important household allergen in U.S. homes. We found that higher mouse allergen levels significantly increased the likelihood of having atopic wheeze and/or asthma symptoms among allergic individuals. Our study extends prior research findings from inner-city populations to the general population. Further research, however, is required to develop and evaluate environmental control measures that cost-effectively reduce and sustain low mouse allergen levels in problem homes. To date, very little information on interventions targeting reductions in residential mouse allergen levels is available (Phipatanakul et al. 2004). We encourage future studies to determine clinically relevant exposure levels for mouse allergen and to provide information on clinical benefits of effective interventions.

## Figures and Tables

**Table 1:** Characteristics of the NSLAH population.

Characteristic	No. (%)^a^
Age (years)	
< 18	762 (26.8)
≥ 18	1,643 (73.2)
Sex	
Male	1,189 (48.2)
Female	1,256 (51.8)
Race	
White	1,788 (79.9)
Black	355 (11.6)
Other	262 (8.5)
Education^b^	
High school level or lower	758 (29.7)
Above high school level	1,646 (70.3)
Living with smoker(s)	
Yes	1,124 (46.0)
No	1,320 (54.0)
Doctor-diagnosed asthma^c^	
Yes	278 (11.2)
No	2,162 (88.8)
Current asthma	
Yes	174 (6.9)
No	2,265 (93.1)
Wheezing in the preceding year	
Yes	353 (15.8)
No	1,966 (84.2)
Wheezing in the preceding month	
Yes	285 (13.0)
No	2,098 (87.0)
Doctor-diagnosed allergies^c^	
Yes	568 (28.1)
No	1,558 (71.9)
Doctor-diagnosed hay fever^c^	
Yes	309 (16.2)
No	1,767 (83.8)

a Weighted for the multistage sampling design of the NSLAH.

b Highest education level attained in the household.

c Lifetime diagnosis.

**Table 2:** Prevalence of asthma and allergy-related outcomes and MUP^a^ levels in the home.

	MUP ≤ 1.6 μg/g		MUP > 1.6 μg/g		
Outcome	No.	% (SE)	No.	% (SE)	*p*-Value^b^
Doctor-diagnosed asthma	164	10.2 (1.0)	114	13.5 (2.0)	0.11
Current asthma	96	6.0 (0.8)	78	8.9 (1.4)	0.05
Wheezing in the preceding year	228	15.7 (1.5)	125	16.6 (2.0)	0.73
Wheezing in the preceding month	171	12.0 (1.6)	114	15.4 (2.3)	0.29
Doctor-diagnosed allergies	368	28.6 (1.8)	197	27.4 (3.0)	0.73
Doctor-diagnosed hay fever	200	16.2 (1.4)	109	16.6 (2.6)	0.88

a MUP concentrations were dichotomized into high and low levels; concentration was considered high if allergen concentration exceeded the cut point value in any of the sampling locations.

b Chi square statistics.

**Table 3:** Current asthma in relation to MUP concentration in the household, all subjects.

Logistic models (*n* = 2,028)	OR (95% CI)	
	MUP ≤ 1.6 μg/g	MUP > 1.6 μg/g
Unadjusted model	1.00	1.41 (0.96–2.06)
Adjusted model^a^	1.00	1.40 (0.94–2.10)

a Adjusted for age, sex, race, education, smoking, and survey season.

**Table 4:** Current asthma in relation to MUP concentration in the household, stratified by atopic status.^a^

	OR (95% CI)		
Logistic models (*n* = 2,028)	Nonatopic	Atopic	*p*-Value for interaction
Unadjusted model	0.74 (0.35–1.59)	2.04 (1.22–3.43)	0.06
Adjusted model^b^	0.69 (0.33–1.44)	1.93 (1.14–3.27)	0.05
Including other allergens			
Fel d 1	0.69 (0.33–1.44)	1.92 (1.13–3.24)	0.05
Can f 1	0.68 (0.32–1.43)	1.87 (1.10–3.21)	0.06
Der f 1^c^	0.68 (0.32–1.42)	1.91 (1.13–3.28)	0.05
Der p 1	0.67 (0.32–1.39)	1.95 (1.15–3.33)	0.04
Bla g 1	0.72 (0.34–1.49)	2.02 (1.18–3.45)	0.05
*Alternaria alternatac*	0.65 (0.31–1.35)	1.79 (1.04–3.07)	0.06
Including endotoxin^c^	0.65 (0.30–1.43)	1.79 (1.04–3.07)	0.07
Including dust weight	0.67 (0.32–1.41)	1.90 (1.12–3.23)	0.05

a Atopy assessed by reported doctor-diagnosed allergies; current asthma/no current asthma = 41/1,448 for nonatopic individuals, 126/413 for atopic individuals; reference group is MUP ≤ 1.6 μg/g.

b Adjusted for age, sex, race, education, smoking, and survey season.

c Because of missing observations, these models include fewer observations than the other adjusted models.
